# Draft genome sequences of three *Xanthomonas translucens* pathovar reference strains (pv. *arrhenatheri*, pv. *poae* and pv. *phlei*) with different specificities for forage grasses

**DOI:** 10.1186/s40793-016-0170-x

**Published:** 2016-08-17

**Authors:** Lena Hersemann, Daniel Wibberg, Franco Widmer, Frank-Jörg Vorhölter, Roland Kölliker

**Affiliations:** 1 Molecular Ecology, Institute for Sustainability Sciences, Agroscope, Zurich Switzerland; 2 Center for Biotechnology, Bielefeld University, Bielefeld, Germany

**Keywords:** Plant pathogen, Bacterial wilt, *hrp* genes, Effector genes, LPS gene cluster, NRPS

## Abstract

As causal agents of bacterial wilt in pastures and meadows, bacteria of the species *Xanthomonas translucens* are a serious issue in forage grass production. So far, only little is known about host-pathogen interactions at the molecular level and the lack of comprehensive genome data impeded targeted breeding strategies towards resistant forage grass cultivars. Here we announce the draft genome sequences of three grass-pathogenic *Xanthomonas translucens* pathotype strains, i.e. pv. *arrhenatheri* LMG 727, pv. *poae* LMG 728 and pv. *phlei* LMG 730 isolated from *Arrhenatherum elatius* (L.) P. Beauv. ex J. Presl & C. Presl (Switzerland), *Poa trivialis* L. (Switzerland) and *Phleum pratense* L. (Norway), respectively. The genomes of all three strains revealed a non-canonical type III secretion system and a set of 22 type III effectors as common virulence-related traits. Distinct inter-pathovar differences were observed for the lipopolysaccharide biosynthesis gene cluster and the presence of nonribosomal peptide synthetases.

## Introduction

*Xanthomonas* spp. are known as destructive plant pathogens affecting a variety of important crop plants [1]. In forage grass production, bacterial wilt caused by pathovars of the species *Xanthomonas translucens* is considered to be one of the most important diseases in temperate grassland regions [2]. Characteristic symptoms include withering of leaves and tillers due to pathogen colonization of the vascular system [3, 4]. In addition, chlorotic and later also necrotic lesions can be observed along infected leaves. Affected grass species belong to a variety of different genera including *Lolium* L., *Festuca* L., *Phleum* L., *Poa* L. and *Arrhenatherum* P. Beauv. [2–4]. In the first years after the initial description of bacterial wilt of forage grasses in 1975 in Switzerland [3], pathogens isolated from infected plants were uniformly assigned to *Xanthomonas campestris* pv. *graminis* [5, 6], later reclassified to *Xanthomonas translucens* pv. *graminis* [7]. However, comprehensive studies on host range specificities pointed towards a further differentiation into four different *Xanthomonas translucens* pathovars named pv. *graminis*, pv. *arrhenatheri*, pv. *poae* and pv. *phlei* [4]. While the pathovar *graminis* is characterized by a broad host range including grass species of different genera, the other three *X. translucens* pathovars show distinct host adaptation to the plant species they have been isolated from: *A. elatius* (*X. translucens* pv. *arrhenatheri* LMG 727), *P. trivialis* (*X. translucens* pv. *poae* LMG 728) and *P. pratense* (*X. translucens* pv. *phlei* LMG 730) [4].

The genome data of these host-specialized pathovar reference strains will allow insight into distinct virulence factors involved in host-specific adaption at the molecular level. In combination with the recently sequenced *X. translucens* pv. *graminis* strain *Xtg*29 [8], these data will valuably complement the genome information on *X. translucens* pathovars which are causing bacterial wilt on forage grasses.

## Organism information

### Classification and features

**Fig. 1:**
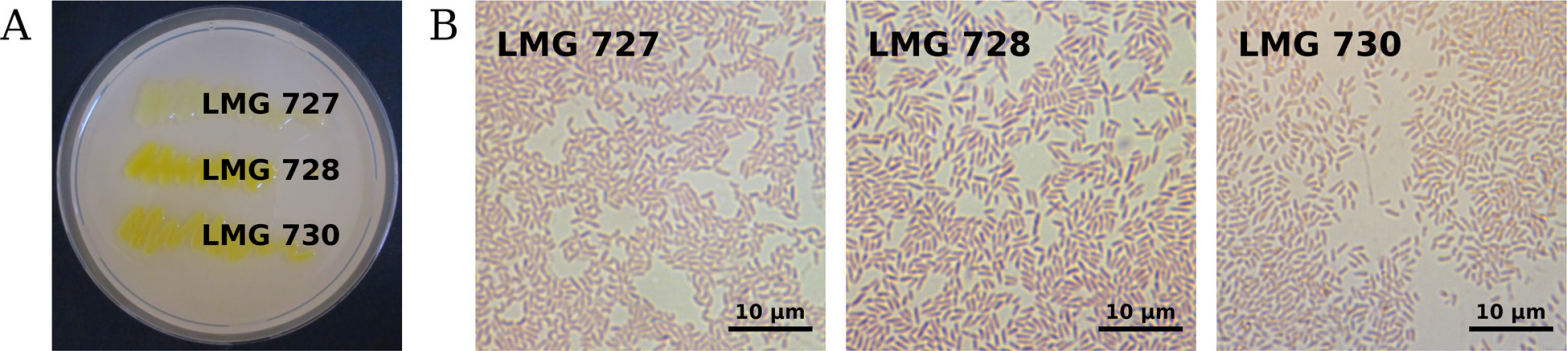
*X. translucens* pv. *arrhenatheri* LMG 727, *X. translucens* pv. *poae* LMG 728 and *X. translucens* pv. *phlei* LMG 730 grown on GYCA medium (**a**) and visualized by light microscopy after over-night cultivation, heat fixation and fuchsine staining (**b**)

*Xanthomonas* spp. are Gram-negative, rod-shaped bacteria, characterized by their typical yellow appearance with varying tones from pale to deep yellow, caused by the pigment xanthomonadin (Fig. 1) [9]. Optimal growth conditions include a temperature of 28 °C and a pH value between 5.5 and 6.5 [10]. For the cultivation of *X. translucens* pathovars, isolated from stalks of infected grasses, GYCA medium containing glucose, yeast extract, CaCO_3_ and agar represents a suitable medium [11, 12]. Further common characteristics of the three pathovar reference strains LMG 727, LMG 728 and LMG 730 are summarized in Table 1.

**Table 1:** Classification and general features of *X. translucens* pv. *arrhenatheri* LMG 727, *X. translucens* pv. *poae* LMG 728 and *X. translucens* pv. *phlei* LMG 730 according to MIGS recommendations [15]

MIGS ID	Property	Term	Evidence code^a^
	Classification	Domain *Bacteria*	TAS [53]
		Phylum *Proteobacteria*	TAS [54]
		Class *Gammaproteobacteria*	TAS [55, 56]
		Order *Xanthomonadales*	TAS [56, 57]
		Family *Xanthomonadaceae*	TAS [56, 58]
		Genus *Xanthomonas*	TAS [59, 60]
		Species *Xanthomonas translucens*	TAS [7]
		Pathovar *arrhenatheri* Strain: LMG 727	TAS [61]
		Pathovar *poae* Strain: LMG 728	TAS [61]
		Pathovar *phlei* Strain: LMG730	TAS [61]
	Gram stain	Negative	TAS [9, 10]
	Cell shape	Rod-shaped	TAS [9]
	Motility	Motile	IDA
	Sporulation	Non-sporulating	TAS [9]
	Temperature range	10–35 °C	NAS
	Optimum temperature	28 °C	TAS [9]
	pH range; Optimum	5.5–6.5	TAS [9, 10]
	Carbon source	D-glucose, D-mannose, sucrose, trehalose, cellobiose, D-fructose	TAS [10]
MIGS-6	Habitat	Plant-associated	TAS [4]
MIGS-6.3	Salinity	Tolerance to 1–2 % NaCl	TAS [10]
MIGS-22	Oxygen requirement	Aerobic	TAS [9, 10]
MIGS-15	Biotic relationship	Parasitic	TAS [4]
MIGS-14	Pathogenicity	Pathogenic	TAS [4]
MIGS-4	Geographic location	LMG 727: Switzerland	TAS [10]
		LMG 728: Switzerland	TAS [10]
		LMG 730: Norway	TAS [10]
MIGS-5	Sample collection	LMG 727: 1978	TAS [10]
		LMG 728: 1978	TAS [10]
		LMG 730: 1978	TAS [10]
MIGS-4.1	Latitude	Not reported	
MIGS-4.2	Longitude	Not reported	
MIGS-4.4	Altitude	Not reported	

^a^ Evidence codes - *IDA* inferred from direct assay, *TAS* traceable author statement (i.e., a direct report exists in the literature), *NAS* non-traceable author statement (i.e., not directly observed for the living, isolated sample, but based on a generally accepted property for the species, or anecdotal evidence). These evidence codes are from the Gene Ontology project [62]

**Fig. 2:**
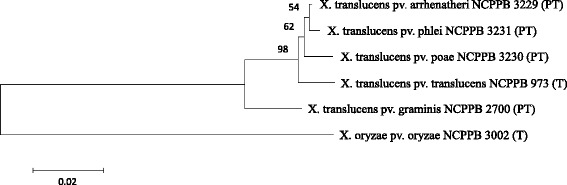
Phylogenetic tree based on partial *gyrB* sequences using the neighbor-joining method with 1,000 bootstrap resampling and calculated with MEGA version 6 [63]. The analysis included type strains (T) and pathotype strains (PT) of the genus *Xanthomonas* listed with their culture collection numbers

Figure 2 shows the phylogenetic position of the three forage grass affecting *Xanthomonas translucens* pathovar reference strains based on a partial *gyrB* DNA sequence of 530 bp [13, 14]. For comparison, the type strain NCPPB 3002 of the rice-affecting species *Xanthomonas oryzae* was used.

## Genome sequencing information

### Genome project history

The strains LMG 727, LMG 728 and LMG 730 were selected for sequencing based on their distinct differences in host range specificities on forage grasses. The whole-genome shotgun projects have been deposited in DDBJ/EMBL/GenBank under the accession numbers CXOI01000001-CXOI01000089 (LMG 727), CXOK01000001-CXOK01000190 (LMG 728) and CXOJ01000001-CXOJ01000142 (LMG 730). Table 2 presents the project information and its association with MIGS version 2.0 compliance [15].

**Table 2:** Project information

MIGS ID	Property	LMG 727	LMG 728	LMG 730
MIGS 31	Finishing quality	High quality draft	High quality draft	High quality draft
MIGS-28	Libraries used	One Paired-end	One Paired-end	One Paired-end
MIGS 29	Sequencing platforms	Illumina MiSeq	Illumina MiSeq	Illumina MiSeq
MIGS 31.2	Fold coverage	109×	249×	315×
MIGS 30	Assemblers	Newbler 2.8	Newbler 2.8	Newbler 2.8
MIGS 32	Gene calling method	Prodigal	Prodigal	Prodigal
	Locus Tag	XTALMG727	XTPLMG728	XTPLMG730
	Genbank ID	CXOI00000000	CXOK00000000	CXOJ00000000
	GenBank Date of Release	2015/08/14	2015/08/14	2015/08/14
	GOLD ID	Gs0118809	Gs0118809	Gs0118809
	BIOPROJECT	PRJEB9902	PRJEB9904	PRJEB9905
MIGS 13	Source Material Identifier	LMG727	LMG728	LMG730
	Project relevance	Study of grassland pathogens		

### Growth conditions and genomic DNA preparation

All three strains were obtained from the BCCM/LMG culture collection of the Laboratory of Microbiology, Ghent University in Belgium (accession numbers: LMG 727, LMG 728 and LMG 730). The strains were grown for 15–20 h in CircleGrow® broth (MP Biomedicals, Santa Ana, USA) at 28 °C and 200 rpm. Genomic DNA was extracted following the protocol for isolation of bacterial genomic DNA using CTAB [16] without the lysozyme application and the subsequent incubation step at 37 °C. The quality of genomic DNA was assessed by gel-electrophoresis and the quantity was estimated by a fluorescence-based method using the Quant-iT PicoGreen dsDNA kit (Invitrogen, Carlsbad, USA) and the Tecan Infinite 200 Microplate Reader (Tecan Deutschland GmbH, Crailsheim, Germany).

### Genome sequencing and assembly

A total of 4 μg genomic DNA of each isolate was used to construct a paired-end sequencing library (TruSeq DNA LT Sample Prep Kit, Illumina Inc., San Diego, USA), which was sequenced applying the paired-end protocol on an Illumina MiSeq system. Upon sequencing and processing of the raw data, a *de novo* assembly was performed using the GS De Novo Assembler software version 2.8. with default settings. The *de novo* assemblies yielded 58 scaffolds (89 contigs) for LMG 727, 129 scaffolds (190 contigs) for LMG 728 and 84 scaffolds (142 contigs) for LMG 730, respectively.

### Genome annotation

Initially, automatic gene prediction and annotation were performed using the genome annotation system GenDB 2.0 [17] and the gene identification strategy Prodigal [18]. Putative rRNA and tRNA genes were identified with RNAmmer [19] and tRNAscan-SE [20]. An automatic annotation was computed based on results of the following different tools: similarity searches were performed against different databases including SWISS-PROT [21], KEGG [22], Pfam [23], TIGRFAM [24] and InterPro [25]. Additionally, SignalP [26] and TMHMM [27] were applied. Finally, the coding sequences were functionally classified by assigning a Cluster of Orthologous Groups number and its corresponding COG category [28] and Gene Ontology numbers [29]. CRISPR repeats were examined using the CRISPR recognition tool [30].

### Genome properties

Whole genome sequencing of the strains LMG 727, LMG 728 and LMG 730 resulted in 109, 249 and 315 fold coverage. Annotation of the 4.76, 4.61 and 4.40 Mb genomes featuring a GC content of 68.31 to 68.37 % was performed within the GenDB 2.0 system and resulted in the prediction of 3,878, 3,851 and 3,749 coding sequences, as well as the following numbers of RNA genes: 55 (3 rRNA genes and 52 tRNA genes), 55 (4 rRNA genes and 51 tRNA genes) and 54 (3 rRNA genes and 51 tRNA genes) for the strains LMG 727, LMG 728 and LMG 730. A total of 15 additional genome features were recorded (Table 3) and the distribution of genes into COG functional categories is presented in Table 4.

**Table 3:** Genome statistics

Attribute	LMG 727	%	LMG 728	%	LMG 730	%
Genome size (bp)	4,754,971	100.00	4,610,480	100.00	4,399,523	100.00
DNA coding (bp)	4,132,338	86.90	3,961,227	85.91	3,805,731	86.50
DNA G+C (bp)	3,250,022	68.35	3,149,419	68.31	3,007,954	68.37
DNA scaffolds	58	100.00	129	100.00	84	100.00
Total genes	3,933	100.00	3,906	100.00	3,803	100.00
Protein coding genes	3,878	98.6	3,851	98.6	3,749	98.6
RNA genes	55	1.40	55	1.40	54	1.40
Pseudo genes	0.00	0.00	0.00	0.00	0.00	0.00
Genes in internal clusters	978	24.86	924	23.65	876	23.03
Genes with function prediction	2,781	70.7	2,759	70.63	2,697	70.91
Genes assigned to COGs	2,987	75.94	2,935	75.14	2,928	76.99
Genes with Pfam domains	3,045	77.42	2,984	76.39	2,968	78.04
Genes with signal peptides	585	14.87	586	15	553	14.54
Genes with transmembrane helices	954	24.25	935	23.93	918	24.13
CRISPR repeats	0.00	0.00	0.00	0.00	0.00	0.00

**Table 4:** Number of genes associated with general COG functional categories

Code	LMG 727	%	LMG 728	%	LMG 730	%	Description
J	173	4.46	173	4.49	171	4.56	Translation, ribosomal structure and biogenesis
A	2	0.00	2	0.00	3	0.00	RNA processing and modification
K	219	5.56	205	5.32	204	5.44	Transcription
L	136	3.5	136	3.53	135	3.60	Replication, recombination and repair
B	1	0.00	1	0.00	1	0.00	Chromatin structure and dynamics
D	33	0.85	32	0.83	33	0.88	Cell cycle control, Cell division, chromosome partitioning
V	71	1.83	62	1.6	70	1.86	Defense mechanisms
T	286	7.37	269	6.98	279	7.44	Signal transduction mechanisms
M	240	6.18	239	6.2	231	6.16	Cell wall/membrane biogenesis
N	124	3.19	126	3.27	122	3.25	Cell motility
U	123	3.17	116	3.01	123	3.28	Intracellular trafficking and secretion
O	0.00	0.00	0.00	0.00	0.00	0.00	Posttranslational modification, protein turnover, chaperones
C	195	5.02	195	5.06	191	5.09	Energy production and conversion
G	211	5.44	214	5.55	209	5.57	Carbohydrate transport and metabolism
E	248	6.39	244	6.33	249	6.64	Amino acid transport and metabolism
F	75	1.93	76	1.97	72	1.92	Nucleotide transport and metabolism
H	147	3.79	141	3.66	143	3.81	Coenzyme transport and metabolism
I	141	3.63	134	3.47	135	3.60	Lipid transport and metabolism
P	196	5.05	198	5.14	189	5.04	Inorganic ion transport and metabolism
Q	147	3.79	76	1.97	75	2.00	Secondary metabolites biosynthesis, transport and catabolism
R	369	9.51	364	9.45	356	9.49	General function prediction only
S	315	8.12	316	8.2	319	8.50	Function unknown
-	891	22.97	916	23.78	821	21.89	Not in COGs

## Extended insights

### Analysis of the type III effector repertoire

Type III effector proteins (T3Es) represent important virulence factors which facilitate successful host colonization by interfering with plant defense mechanisms [31]. Vice versa, effector proteins are able to trigger defense responses if recognized by corresponding resistance genes within the plant [32]. Thus, effector proteins are considered as important candidate genes, contributing to host range specificity [33]. In order to identify T3Es within the genome data of the three *X. translucens* pathotype strains LMG 727, LMG 728 and LMG 730, their corresponding CDS were compared against publicly available effector protein sequences [34]. An e-value of 1E-15 was used as a threshold for identifying putative T3Es. Additionally, the presence of plant-inducible promoter boxes has been identified as described recently [8]. Genes with upstream PIP-boxes were analyzed by applying Blastx against the non-redundant protein sequences (nr) database [35]. Table 5 represents a list of putative type III effector proteins and corresponding *Xanthomonas* effector classes [36] identified for LMG 727, LMG 728 and LMG 730. Listed percentage identities and e-values refer to the lowest values obtained in Blastp analysis within the homologous CDS of the three pathovar reference strains. Analysis of the effector repertoire revealed the presence of 30, 31 and 29 putative T3Es in the genome data of LMG 727, LMG 728 and LMG 730, respectively. Twenty-two putative effector proteins were conserved among all three pathotype strains and 21 of those could clearly be assigned to one of the known effector classes of the genus *Xanthomonas*. Furthermore, one, three and five homologues of transcription activator like effectors (TALEs) [37] have been identified for LMG 730, LMG 727 and LMG 728, respectively, and may be worth deeper analysis.

**Table 5:** Homologues of type III effector proteins

Effector class^a^	LMG 727^b^	LMG 728^b^	LMG 730^b^	Identity (%)	E-value
AvrBs2	XTALMG727_3766	XTPLMG728_3304	XTPLMG730_3385	92.04	0.0
	XTALMG727_3767	XTPLMG728_3305	XTPLMG730_3384	89.37	0.0
XopAA	XTALMG727_0004*	XTPLMG728_1109*	XTPLMG730_1729	79.86	0.0
	N	XTPLMG728_0423	N		
	N	XTPLMG728_0424	N		
	N	XTPLMG728_0425	N		
	N	XTPLMG728_0426*	N		
XopAD	XTALMG727_0614	XTPLMG728_3670	XTPLMG730_1368	91.65	0.0
	XTALMG727_1307	N	N		
XopAH	N	N	XTPLMG730_1645*		
XopB	XTALMG727_0958*	XTPLMG728_0265*	XTPLMG730_1037	83.91	0.0
	N	N	XTPLMG730_1038*		
XopC	XTALMG727_2735	XTPLMG728_0929	XTPLMG730_2930	93.06	0.0
XopE	N	N	XTPLMG730_2339*		
XopF	XTALMG727_0160	XTPLMG728_3393*	XTPLMG730_0026*	95.18	0.0
	XTALMG727_0243	XTPLMG728_2858*	N	85.88	0.0
	XTALMG727_0242*	N	N		
XopG	XTALMG727_1016	XTPLMG728_2920	XTPLMG730_2662	75.58	3E-111
XopH	XTALMG727_1259	N	N		
XopI	XTALMG727_3409	N	XTPLMG730_3626	89.28	0.0
XopJ	XTALMG727_3363	N	N		
	XTALMG727_3364*	N	N		
XopK	XTALMG727_1234*	XTPLMG728_3296*	XTPLMG730_2968*	95.06	0.0
XopL	XTALMG727_3597*	XTPLMG728_2315*	XTPLMG730_2526*	81.55	0.0
	XTALMG727_3852*	XTPLMG728_3529*	XTPLMG730_3754*	58.94	1E-176
	N	N	XTPLMG730_3767*		
XopN	XTALMG727_1719	XTPLMG728_0715	XTPLMG730_2395	92.85	0.0
XopP	XTALMG727_0476*	XTPLMG728_1678*	XTPLMG730_0352*	90.97	0.0
	XTALMG727_1884*	XTPLMG728_2570*	XTPLMG730_3584*	86.94	0.0
	N	XTPLMG728_3453	XTPLMG730_0007	93.01	2E-125
	N	XTPLMG728_3824*	N		
XopQ	XTALMG727_3080*	XTPLMG728_1854*	XTPLMG730_2816*	97.69	0.0
XopR	XTALMG727_2355	XTPLMG728_1451	XTPLMG730_1831	83.78	3E-124
XopV	XTALMG727_0112*	XTPLMG728_3243*	XTPLMG730_2612*	90.94	0.0
XopX	XTALMG727_1509	XTPLMG728_1839	XTPLMG730_0225	85.66	0.0
	XTALMG727_2734	XTPLMG728_0930	XTPLMG730_2929	90.32	0.0
XopZ	XTALMG727_0656	XTPLMG728_3741	XTPLMG730_1408	97.52	0.0
XopAM	XTALMG727_2702*	XTPLMG728_2341*	XTPLMG730_3036*	96.69	0.0
XopAF	N	XTPLMG728_1151	N		
AvrXccA	N	XTPLMG728_0197	XTPLMG730_0963	96.77	0.0
-	XTALMG727_0041*	XTPLMG728_0463*	XTPLMG730_1692*	87.88	3E-149
-	XTALMG727_1653*	N	N		

^a^Assigned effector classes based on sequence homology to T3Es listed in the publication of White et al. [36] and publically accessible data [34]

^b^Identified effector proteins are listed by the corresponding locus tags, while N indicates, that no homologous effector protein could be identified. The presence of PIP-boxes is indicated by asterisks (*)

The secretion of T3Es is mediated by the type III secretion system [38]. All three sequenced strains (LMG 727, LMG 728, LMG 730) carried a hypersensitivity response and pathogenicity (*hrp*) gene cluster homologous to the non-canonical type III secretion system recently published as a prevalent finding in the genome of *Xanthomonas translucens* pv. *graminis* strain *Xtg*29 [8]. However, in the genome of LMG 728 the neighboring genes *hrcC* and *hrpX* were more distantly located to the main part of the *hrp* gene cluster.

### Characterization of further virulence-related traits

In addition to the type III secretion of effector proteins, a wide range of additional mechanisms contribute to bacterial virulence. In the early stages of pathogenesis, flagellar-mediated motility represents a prevalent mechanism for invasion of the host plant [39]. The presence of a flagellar gene cluster [40] was common for LMG 727, LMG 728 and LMG 730 and confirmed by the motility observed for these three strains (data not shown). Successful host colonization is further depending on type I and type II secretion systems which are involved in the translocation of virulence factors (i.e. toxins and degradative enzymes) and thus substantially contribute to bacterial virulence [41]. A T2SS encoding *xps* gene cluster [42] as well as the corresponding *rax* genes of the T1SS [43] were identified for the pathovar reference strains LMG 727, LMG 728 and LMG 730. Furthermore, in all three genomes homology to the chromosomal type IV secretion system gene cluster of *X. axonopodis* pv. *citri* [44] was observed; however, a corresponding *virB7* homologue could not be identified in any of the strains.

Whereas all of the above mentioned gene clusters were found to be highly conserved among the three pathovar reference strains, we observed a significant difference for the O-antigen encoding part of the lipopolysaccharide (LPS) gene cluster [45]. While the flanking genes of this region, i.e. *etfA* and *metC* are widely conserved among *Xanthomonas* spp. a highly divergent gene content has been reported for the interjacent region [46, 47]. Comparison of the corresponding region of LMG 727, LMG 728 and LMG 730 revealed 21, 18 and 19 genes, respectively. Among these, 16 to 17 were homologous across all three genomes. Differences in the number of homologous genes were due to nonsense mutations, which caused gene separation in two individual genes for LMG 727 and LMG 728.

Another inter-pathovar difference was found for the *gum* gene cluster, which encodes for xanthan biosynthesis [48]. This extracellular polysaccharide has recently been shown to be involved in biofilm formation and to promote epiphytic growth on host plants [49, 50]. Both, LMG 728 and LMG 730 possessed 11 *gum* genes as described recently for *X. translucens* pv. *graminis Xtg*29 [8]. Also the LMG 727 genome was found to largely encode the corresponding gene cluster; however, missense mutation of the stop codon in *gumK* resulted in a gene fusion with the neighboring upstream gene *gumL*. Analysis of deletion mutants of both genes in *X. oryzae* pv. *oryzae* revealed only minor effects on xanthan production and no reduced virulence for the *gumL* mutant, while the *gumK* mutant was characterized by both, a reduced xanthan production and impaired virulence on rice leaves [51]. However, considering the mucoid phenotype of LMG 727 (Fig. 1), production of the exopolysaccharide xanthan seemed not impaired by the observed non-stop mutation. Moreover, the *rpf* gene cluster, involved in the regulation of xanthan production and further virulence-related features [52], was identified in the LMG 728 and LMG 730 genomes as well as for LMG 727.

Both, the LPS and xanthan biosynthesis gene clusters, revealed distinct characteristics of the pv. *arrhenatheri* reference strain. Along with these findings, we identified a nonribosomal peptide synthetase gene cluster consisting of 10 genes to be solely present in the LMG 727 genome.

## Conclusions

Aiming to identify virulence factors putatively involved in host range specificity of forage grass pathogens, we sequenced three pathovar reference strains of the species *Xanthomonas translucens*. In a first step, we analyzed the strain-specific type III effector repertoires, which indicated clear inter-pathovar differences along with a subset of effector proteins highly conserved among all three strains. Accordingly, high conformity in gene content and sequence homology were identified for four secretion systems and the flagellar gene cluster, whereas all three genomes were found to be characterized by a divergent gene cluster of LPS biosynthesis when compared to each other. With regard to the deviating gene content of the *gum* gene cluster and the identified NRPS genes, the pv. *arrhenatheri* reference strain LMG 727 revealed further pathovar-specific characteristics. Altogether, these data sets represent a useful basis for the functional analysis of distinct genomic traits involved in host range adaptation of *X. translucens* pathovars and a valuable resource for future breeding strategies towards resistant forage grass cultivars.

## Abbreviations

CDS: Coding sequence
CTAB: Cetyltrimethylammonium bromide
LPS: Lipopolysaccharide
NRPS: Non-ribosomal peptide synthetase
PIP: Plant-inducible promoter
T1SS: Type I secretion system
T2SS: Type II secretion system
T3Es: Type III effector proteins
TALEs: Transcription activator-like effectors
